# Functional analysis of the C-reactive protein (CRP) gene -717A>G polymorphism associated with coronary heart disease

**DOI:** 10.1186/1471-2350-10-73

**Published:** 2009-07-22

**Authors:** Laiyuan Wang, Xiangfeng Lu, Yun Li, Hongfan Li, Shufeng Chen, Dongfeng Gu

**Affiliations:** 1 Department of Evidence Based Medicine & Division of Population Genetics, Fu Wai Hospital & Cardiovascular Institute, Chinese Academy of Medical Sciences & Peking Union Medical College, Beijing, PR China; 2 National Human Genome Center at Beijing, Beijing, PR China

## Abstract

**Background:**

Atherosclerosis underlies the major pathophysiological mechanisms of coronary heart disease (CHD), and inflammation contributes to all phases of atherosclerosis. C-reactive protein (CRP), a sensitive, but nonspecific marker of inflammation has been shown to play proatherogenic roles in the process of atherosclerosis. Our previous report showed that rs2794521 (-717A>G), located in the promoter of the CRP gene, was independently associated with CHD in Chinese subjects. In the present study, we tried to investigate the biological significance of this genetic variation *in vitro*.

**Methods:**

The influence of G to A substitution at the site of rs2794521 on the transcriptional activity of the promoter of the CRP gene was assessed by luciferase reporter assay, and protein binding to the site of rs2794521 was detected by EMSA assay.

**Results:**

The G to A exchange at the site of rs2794521 resulted in an increased transcriptional activity of the promoter of CRP gene, and glucocorticoid receptor (GR) protein factor bound drastically differently to the A and G alleles at the site of rs2794521.

**Conclusion:**

These results provided functional evidence supporting the association of the SNP rs2794521 of the CRP gene with CHD probably through regulating the expression level of CRP by different variations of rs2794521.

## Background

The global burden of cardiovascular disease (CVD) represents the highest cause of mortality and one of the highest causes of morbidity both in high-income and low/middle-income countries[1,2]. More than 7 million of the almost 17 million cardiovascular deaths each year is a result of coronary heart disease (CHD). According to a report issued by the World Health Organization, CHD is the leading cause of death in men and women aged ≥60 years. Prevailing evidence has shown that CHD is a complex disease where both multiple minor genetic and environmental factors interact [3].

Atherosclerosis underlies the major pahthophysiological mechanisms of CHD[4]. Originally considered a disorder of lipid metabolism, atherosclerosis is now regarded as an inflammatory disease [5-7]. Inflammation contributes to all phases of atherosclerosis, from fatty streak initiation to cardiovascular disease events. Researches into the inflammatory nature of atherosclerosis suggested that inflammatory-response proteins, particularly acute-phase reactants, might be involved in the pathogenesis of developing atherosclerosis. C-reactive protein (CRP), a sensitive, but nonspecific marker of inflammation (an acute-phase reactant) [8] has been shown to play proatherogenic roles in the process of atherosclerosis via effects on monocytes and endothelial cells: CRP directly induced the expression of adhesion molecules and monocyte chemoattractant protein-1 (MCP-1) by endothelial cells [9,10], increased plasminogen activator inhibitor-1 expression and activity in endothelial cells [11], decreased eNOS mRNA, protein abundance and enzyme activity in endothelial cells, and preincubation of cells with CRP also significantly increased the adhesion of monocytes to endothelial cells [12]. CRP deposited in the arterial wall in early atherosclerotic lesions [13], and might contribute to the formation of foam cells in atherosclerotic lesions by causing the aggregation of LDL molecules that are then taken up by macrophages through a CD32-independent pathway [14]. Family and twin studies have estimated that genetic factors accounted for 35–40% of the variance of CRP levels [15-17], and polymorphisms in the CRP gene have consistently been associated with basal CRP levels in both men and women[18,19]. Genetic variants within the CRP gene are related to the observed CRP response during and after acute coronary syndromes [20]or acute ischemic stroke/TIA[21].

Our previous report showed that rs2794521 (-717A>G) located in the promoter of the CRP gene was independently associated with CHD in Chinese subjects[4], the frequency of A allele carriers was significantly higher in patients than in controls, and individuals carrying A allele had an approx. 6.8-fold higher risk of developing CHD compared with those not carrying this allele. However, the influence of G to A substitution at the site of rs2794521 on the transcriptional activity of the promoter of the CRP gene is unknown, in particular a difference for nuclear proteins binding to A or G allele at the site of rs2794521 has not been studied. In the present study, the biological relevance of the SNP rs2794521 significantly associated with CHD in Chinese subjects[4] was studied by various functional assays *in vitro*.

## Methods

### Construction of luciferase reporter gene

The genomic sequence of the CRP gene containing the site of rs2794521 was amplified by PCR from 1 individual homozygous for the A allele and 1 individual homozygous for the G allele. The PCR primers were tailored to incorporate a Kpn I site at the 5' end and an Xho I site at the 3' end of the amplified fragments. These 915-base pair (bp) genomic fragments, corresponding to nt -858 to +57 of the CRP promoter region, were then purified, digested with the two designated restriction endonucleases, and further subcloned into the Kpn I and Xho I sites of the firefly luciferase expressing pGL3-basic vector (Promega, USA) to create two plasmids: pGL3-basic-A and pGL3-basic-G. Both constructs were sequenced to verify that the only ambiguity was the polymorphic site.

### Cell cultures, transfections and luciferase assays

Human HepG2 cells were grown in Dulbecco's modified Eagle medium (DMEM) medium with 10% fetal bovine serum (FBS), 5% heat-inactivated donor horse serum and 10 μg/ml of penicillin/streptomycin at 37°C and 5% CO_2_. Cells were seeded at a density of 2.5 × 10^5 ^cells per well in a 12-well plate 24 hours prior to transfection in medium without antibiotics. Transfections were performed with Lipofectamine 2000 (Invitrogen, USA) according to the manufacturer's instructions. Approximately 2 μg of the luciferase reporter gene were cotransfected with 0.5 μg of pRL-TK (Renilla luciferase, Promega, USA) as an internal control for variations in transfection efficiency. Transfection using pGL3-basic vector without an insert was used as a negative control. The transfected cells were harvested after 24 hours, and the luciferase activity was measured with Dual-Luciferase Reporter Assay System (Promega, USA) using a luminometer (TD-20, Turner Designs, Sunny vale, USA).

### Preparation of nuclear extracts

Nuclear extracts from HepG2 cells were prepared with NE-PER^@^Nuclear and Cytoplasmic Extraction Reagents kit (Pierce, Rockford, IL, USA) according to the manufacture's instructions. Protein concentrations were determined by a bicinchoninic acid (BCA) assay (Pierce, USA), and the nuclear extracts were stored at -80°C.

### Electrophoretic mobility shift assay and supershift assays

Synthetic double-stranded oligonucleotide probes(A: AACCAAACACCGC***A***TGTTCTCACTC; and G: AACCAAACACCGC***G***TGTTCTCACTC) corresponding to the genomic sequence in the promoter of the CRP gene with either A or G allele at the rs2794521 site were labeled with biotin (Shanghai Sangon Biological Engineering Technology and Services Co., Ltd., Shanghai, China). Electrophoretic mobility shift assays were performed by using the LightShift™ Chemiluminescent EMSA kit (Pierce, USA). For each gel shift reaction (20 μl), a total of 20 fmol biotin-labeled probe was combined with 15 μg nuclear extract prepared from HepG2 cells, 1 μg poly (dI-dC), and 1×binding buffer. For competition assays, a 200-fold molar excess of unlabeled A or G probe was pre-incubated for 5 minutes at room temperature with nuclear extracts before the addition of the labeled probe. For supershift experiments, antibody specific for glucocorticoid receptor (GR) or nonspecific rabbit IgG (Santa Cruz Biotechnology) was used. The antibody or rabbit IgG (2 μg) was incubated with nuclear extract for 1 hr at 4°C, followed by an additional incubation for 20 minutes at room temperature with a labeled probe A. The reaction mixture was resolved on a non-denaturing 6% acrylamide gel in 0.5× TBE buffer, the electrophoresised binding reactions were then transferred to nylon membrane, and cross-linking was performed for 5 minutes with a UV cross linker. The signal of the biotin-labeled DNA bound to the membrane was detected with a LightShift Chemiluminescent EMSA Kit (Pierce, USA) according to the manufacturer's instructions.

### Statistical analysis

Luciferase assay data were assessed by Student's t-test using SigmaPlot (SPSS) and a *P*-value less than 0.05 was considered statistically significant.

## Results

### Allele-specific effect of the rs2794521 on transcriptional activity

**Figure 1:**
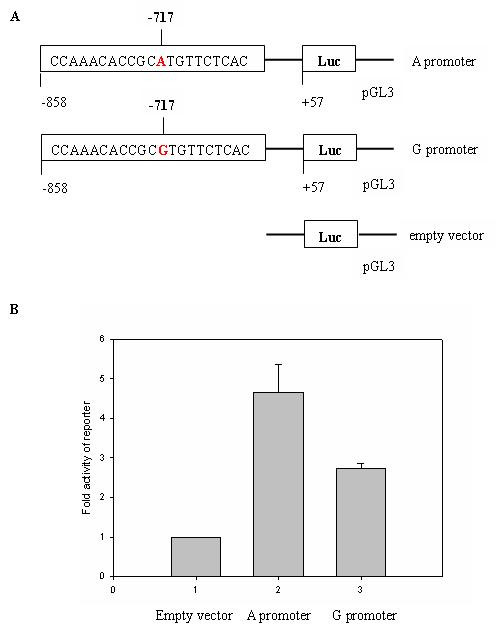
**Allelic change from G (nonsusceptible allele) to A (susceptible allele) at the site of rs2794521 located in the promoter of CRP gene enhanced transcriptional activity of the promoter of CRP in HepG2 cells**. ***A***, schematic representation of the plasmid constructs. 915 bp fragment of CRP promoter (-858/+57) containing either the A or G allele (i.e. A and G promoter, respectively) was inserted in the pGL3-basic luciferase expression vector. Empty vector: pGL3-basic vector. ***B***, HepG2 cells were contransfected with individual firefly luciferase (FLuc)expressing plasmid (A promoter, G promoter, empty vector) and pRL-TK as described in Materials and Methods. Cells were harvested after 24 hours, and relative luciferase activity (FLuc/Renilla luciferase) was determined. Fold luciferase activity of the variant A construct was 1- to 2-fold higher than that of the common G construct (P < 0.005). Data are present as fold-induction compared with promoter vector without insert. Bares are mean ± standard error of 3 different experiments, each performed in dulplicate.

We considered that transcriptional regulation elements might be located in the promoter region of the CRP gene and performed biological assays to investigate whether the rs2794521 SNP located in the promoter of the CRP gene had a direct effect on promoter transcriptional activity. A genomic fragment of the CRP gene carrying either the A or G allele at the site of rs2794521 was inserted into the Hind III and Kpn I sites of the firefly luciferase expressing pGL3-basic vector (Fig. 1A). The activity of these luciferase reporter gene constructs was assessed in transient transfection assays in HepG2 cells. The luciferase activity of the variant A construct was 1- to 2-fold higher than that of the common G construct (P < 0.005; Fig. 1B). This result clearly indicated that transcriptional activity of the promoter of CRP gene containing the A allele was higher than that of the promoter of CRP gene containing the G allele.

### Allele-specific effect of the rs2794521 on binding of nuclear proteins to the promoter of the CRP gene

**Figure 2:**
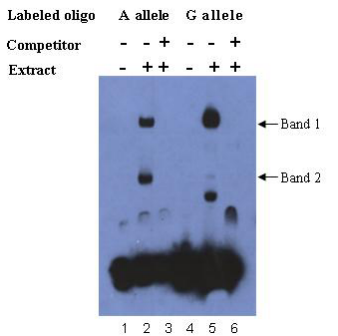
**Alleles of rs2794521 differentially bind transcription factor(s)**. EMSA was performed by using biotin-labeled probe A or probe G with HepG2 cell nuclear extract, with or without competition from unlabeled probe A or probe G. The GR binds to a GRE as a monomer and a dimer as previously reported, and the incubation of nuclear extract of HepG2 cells with probe A showed two kinds of DNA-protein complex with the GR motif oligonucleotide probes, which probably represent monomer (Band 2) and dimer (Band 1) of GR (Lane 2). The incubation of nuclear extract of HepG2 cells with probe G showed a large increase in the dimer (Lane 5, band 1), and a dramatic reduction in monomer (Lane 5, band 2). The experiments were repeated three times with similar results.

To elucidate specific nuclear factors that might bind the disease-susceptible allele, we analyzed the sequence around rs2794521 site using TRANSFAC software, and found that the sequence containing G allele at the site of rs2794521 is a not well-matched GR binding motif, and the G to A exchange at the site of rs2794521 resulted in a well-matched GR binding motif. Therefore, we performed electrophoretic mobility shift assays to determine the nuclear factors that might bind to oligonucleotides corresponding to genomic sequence of the A or G allele of rs2794521, and to determine whether the binding of the transcription factor(s) differed for the A and G alleles. The GR binds to a glucocorticoid response element (GRE) as a monomer and a dimer as previously reported[22], and the incubation of nuclear extract of HepG2 cells with probe A showed two kinds of DNA-protein complex with the GR motif oligonucleotide probes, which probably represent monomer (Band 2) and dimer (Band 1) of GR (Lane 2). The incubation of nuclear extract of HepG2 cells with probe G showed a large increase in the dimer (Lane 5, band 1), and a dramatic reduction in monomer (Lane 5, band 2). These shifted bands could be completely abolished by 200-fold unlabeled A or G probe (Fig. 2, lane 3 and 6), indicating specific binding of nuclear protein(s) to the oligonucleotide corresponding to genomic sequence of A or G allele.

**Figure 3:**
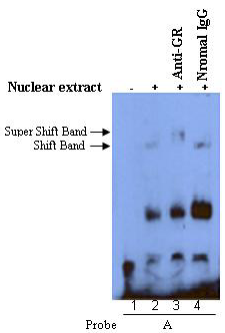
**Supershift assays confirmed the identification of the DNA-binding protein**. Supershift assays with biotin-labeled 25 bp oligonuceotides containing the A allele of rs2794521 and nuclear extracts from HepG2 cells in the presence of anti-GR (Lane 3) or normal IgG (Lane 4). Labeled oligonucleotides incubated without the nuclear extracts were included as negative controls (Lane 1). 2 μg of anti-GR antibody or normal IgG was preincubated with the nuclear extract for 1 hour at 4°C before the labeled probe was added. The experiments were repeated three times with similar results.

Because the G to A exchange at the site of rs2794521 resulted in a well-matched GR binding motif and the GR bound to both the A and G alleles, we further performed supershift assay to confirm the identity of the DNA-binding protein using the probe A and the antibody specific for GR or nonspecific rabbit IgG. The DNA-protein complex was successfully supershifted with the anti-GR antibody (Fig. 3, lane 3), but not the rabbit IgG (Fig. 3, lane 4). Taken together, these results clearly demonstrate that SNP rs2794521 in the promoter of CRP gene is within a GR-binding motif, and the A to G substitution results in different binding affinity for monomer or dimer of GR protein.

## Discussion

It is well known that CHD is a complex disease where both multiple minor genetic and environmental factors interact[3]. Our previous report[4] found that the A allele of rs2794521 located in the promoter region of the CRP gene was independently associated with CHD in Chinese subjects. Individuals carrying the A allele had an approx. 6.8-fold higher risk of developing CHD compared with those not carrying this allele. This finding has been confirmed, as the same SNP was found to correlate with occurrence of myocardial infarction or thromboembolic stroke in the Physician's Health Study[19]. In the present study, we found that transcriptional activity of the promoter of CRP gene containing the A allele was higher than that of the promoter of CRP gene containing the G allele. Among several case-control studies involving the SNP rs2794521 with CHD [4,19,21,23], our present study is the first to confirm that the SNP rs2794521 is to be functional.

E. Ben-Assayag et al. reported that -717A>G was associated with triggered CPR levels during acute stroke/TIA[21]. This study indicated that -717A>G located at the promoter region of the CRP gene was far more than just a marker associated with CHD, but might have native biological function. Our present study showed an increased transcriptional activity of the promoter of the CRP gene driven by A allele, therefore we deduced that the association of -717A>G with altered plasma levels of CRP[21] might be due to the exchange of G to A allele at the site of rs2794521, thus resulting in the increase of the CRP levels during some stimulating conditions. Additional studies aimed to confirm this finding are warranted.

Nuclear protein factor(s) plays important roles in the regulation of gene expression. There is a well-matched GR binding motif at the site of rs2794521 after the exchange of G to A allele. Our EMSA assay found that two kinds of DNA-protein complex with oligonucleotide probe A corresponding to genomic sequence of the A allele, which probably represent monomer (Band 2) and dimer (Band 1) of GR (Lane 2) as previously reported[22], but a large increase in the dimer (Lane 5, band 1), and a dramatic reduction in monomer (Lane 5, band 2) binding to oligonucleotide probe G corresponding to genomic sequence of the G allele. The EMSA experiment showed that the binding of GR to the A and G alleles differed drastically. The supershift EMSA was then performed with only one probe, probe A, because of the higher similarity of probe A sequence to GR binding motif. The supershift EMSA experiment showed that it is GR protein that bound to the A allele of rs2794521. This indicated that the binding of GR factor to the two alleles of rs2794521 was drastically different. But it is unknown whether the different binding of GR to the two alleles of rs2794521 accounts for the different transcriptional activity of the promoter of the CRP gene derived by A or G allele. GR can contribute to the regulation of gene transcription in different ways[24]. Unusually, glucocorticoids bind to the GR, which are translocated to the nucleus and the GR functions as dimer. However, the activated GR may bind to other nuclear receptors forming heterodimers, and the heterodimers bind to GREs or as yet unknown DNA elements thereby affecting the transcription rate of glucocorticoid-responsive genes [24]. According to the reference [22,24] and our results, we deduced that the monomer of GR might bind to the A allele of rs2794521, and affect the transcription activity of the promoter of the CRP gene through the formation of heterodimers with other nucleus receptors, which needs to be further studied.

## Conclusion

In summary, we found that the G to A exchange at the site of rs2794521 resulted in an increased transcriptional activity of the promoter of CRP gene, and the GR protein factor bound drastically differently to the A and G alleles. These molecular data provided functional evidence of association of a common rs2794521 of the CRP gene with CHD in Chinese Han population probably through regulating the expression level of CRP by different variations of rs2794521. Nonetheless, additional research will be required to better define the functional significance of this variant and to clarify the mechanism of the association of inflammatory protein involving the CRP gene with CHD.

## Abbreviations

BCA: bicinchoninic acid; bp: base pair; CHD: coronary heart disease; CVD: cardiovascular disease; CRP: C-reactive protein; EMSA: electrophoretic mobility shift assay; PCR: polymerase chain reaction; SNP: single nucleotide polymorphism.

## Competing interests

The authors declare that they have no competing interests.

## Authors' contributions

LYW and HFL carried out the molecular biological studies. XFL, YL and SF CH performed the statistical analysis. LYW and DFG conceived of the study, and participated in its design and coordination and helped to draft the manuscript. All authors read and approved the final manuscript.

## Pre-publication history

The pre-publication history for this paper can be accessed here:


